# Risk Factors for Development of Acute Kidney Injury in Patients with Urinary Tract Infection

**DOI:** 10.1371/journal.pone.0133835

**Published:** 2015-07-27

**Authors:** Chih-Yen Hsiao, Huang-Yu Yang, Meng-Chang Hsiao, Peir-Haur Hung, Ming-Cheng Wang

**Affiliations:** 1 Department of Internal Medicine, Ditmanson Medical Foundation Chia-yi Christian Hospital, Chia-yi, Taiwan; 2 Department of Hospital and Health Care administration, Chia-Nan University of Pharmacy and Science, Tainan, Taiwan; 3 Department of Nephrology, Chang Gung Memorial Hospital, Chang Gung University, College of Medicine, Taoyuan, Taiwan; 4 Department of Genetics, University of Alabama at Birmingham, Birmingham, Alabama, United States of America; 5 Department of Applied Life Science and Health, Chia-Nan University of Pharmacy and Science, Tainan, Taiwan; 6 Division of Nephrology, Department of Internal Medicine, National Cheng Kung University Hospital, College of Medicine, National Cheng Kung University, Tainan, Taiwan; 7 Bloomberg School of Public Health, Johns Hopkins University, Baltimore, MD, 21205, United States of America; University of Toledo, UNITED STATES

## Abstract

Acute kidney injury (AKI) is associated with high morbidity and mortality. Urinary tract infection (UTI) may be associated with sepsis or septic shock, and cause sudden deterioration of renal function. This study investigated the clinical characteristics and change of renal function to identify the risk factors for development of AKI in UTI patients. This retrospective study was conducted in a tertiary referral center. From January 2006 to January 2013, a total of 790 UTI patients necessitating hospital admission were included for final analysis. Their demographic and clinical characteristics and comorbidities were collected and compared. Multivariate logistic regression analysis was performed to evaluate the risk factors for AKI in UTI patients. There were 97 (12.3%) patients developing AKI during hospitalization. Multivariate logistic regression analysis showed that patients with older age (OR 1.02, 95% CI 1.00–1.04, *P* = 0.04), diabetes mellitus (DM) (OR 2.23, 95% CI 1.35–3.68, *P* = 0002), upper UTI (OR 2.63, 95% CI 1.53–4.56, *P* = 0001), afebrile during hospitalization (OR 1.71, 95% CI 1.04–2.83, *P* = 0036) and lower baseline eGFR [baseline eGFR 45–59 mL/min/1.73 m^2^ (OR 2.12, 95% CI 1.12–4.04, *P* = 0.022), baseline eGFR 30-44 mL/min/1.73 m^2^ (OR 4.44, 95% CI 2.30–8.60 *P* < 0.001) baseline eGFR < 30 mL/min/1.73 m^2^ (OR 4.72, 95% CI 2.13–10.45, *P* <0.001), respectively] were associated with increased risk for development of AKI. were associated with increased risk for development of AKI. Physicians should pay attention to UTI patients at risk of AKI (advancing age, DM, upper UTI, afebrile, and impaired baseline renal function).

## Introduction

Urinary tract infection (UTI) is one of the most common bacterial infections [1]. The overall annual incidence of UTI was 1.75% among residents of the Calgary Health Region, Canada during 2004–2005 [2]. The 2007 National Ambulatory Medical Care Surveys estimated UTIs were responsible for nearly two million visits to emergency departments annually in the USA [3].

UTI can be either asymptomatic or symptomatic, characterized by a wide spectrum of symptoms ranging from mild irritative voiding to bacteremia, sepsis, shock or even death. In specific patient groups, urosepsis may show high mortality rates of 25% to 60% [4].

Sepsis is one of the most common triggers of acute kidney injury (AKI) [5], and about 60% patients with septic shock developed AKI [6]. Acute UTI may cause sudden deterioration of renal function, especially for urinary tract obstruction [7]. AKI is associated with high morbidity and mortality during acute care [8–10]. Patients with severe AKI were under increased risk of end stage renal disease and even death after hospital discharge [11–13].

Since sepsis-related AKI leads to poor outcomes and increased healthcare costs, identification of the risk factors for development of AKI in patients with UTI is critical. However, there were few studies focusing on this important issue. In this retrospective study, we investigated the clinical characteristics and change of renal function to identify the risk factors for development of AKI in UTI patients.

## Materials and Methods

### Ethics statement

This retrospective observational study complied with the guidelines of the Declaration of Helsinki and was approved by the Medical Ethics Committee of Chia-Yi Christian Hospital, a tertiary referral center located in the southwestern part of Taiwan. Since this study involved retrospective review of existing data, approval from the Institutional Review Board of Chia-Yi Christian Hospital was obtained (Approval # CYCH-IRB-100015), but without specific informed consent from patients. Furthermore, not only were all data securely protected (by delinking identifying information from the main data sets) and made available only to investigators, but they were also analyzed anonymously. The Institutional Review Board of Chia-Yi Christian Hospital specifically waived the need for consent for these studies. Finally, all primary data were collected according to procedures outlined in epidemiology guidelines to strengthen the reporting of observational studies.

### Study Conduct

This retrospective study was conducted in a tertiary referral center located in a city of southern Taiwan with a population of 547,000 people. The hospital has 1,000 acute care beds, and serves approximately 3,800 outpatients and 260 emergency patients daily. The authors had full access to the results and vouch for the completeness and accuracy of the data and analysis.

### Study population

**Fig 1:**
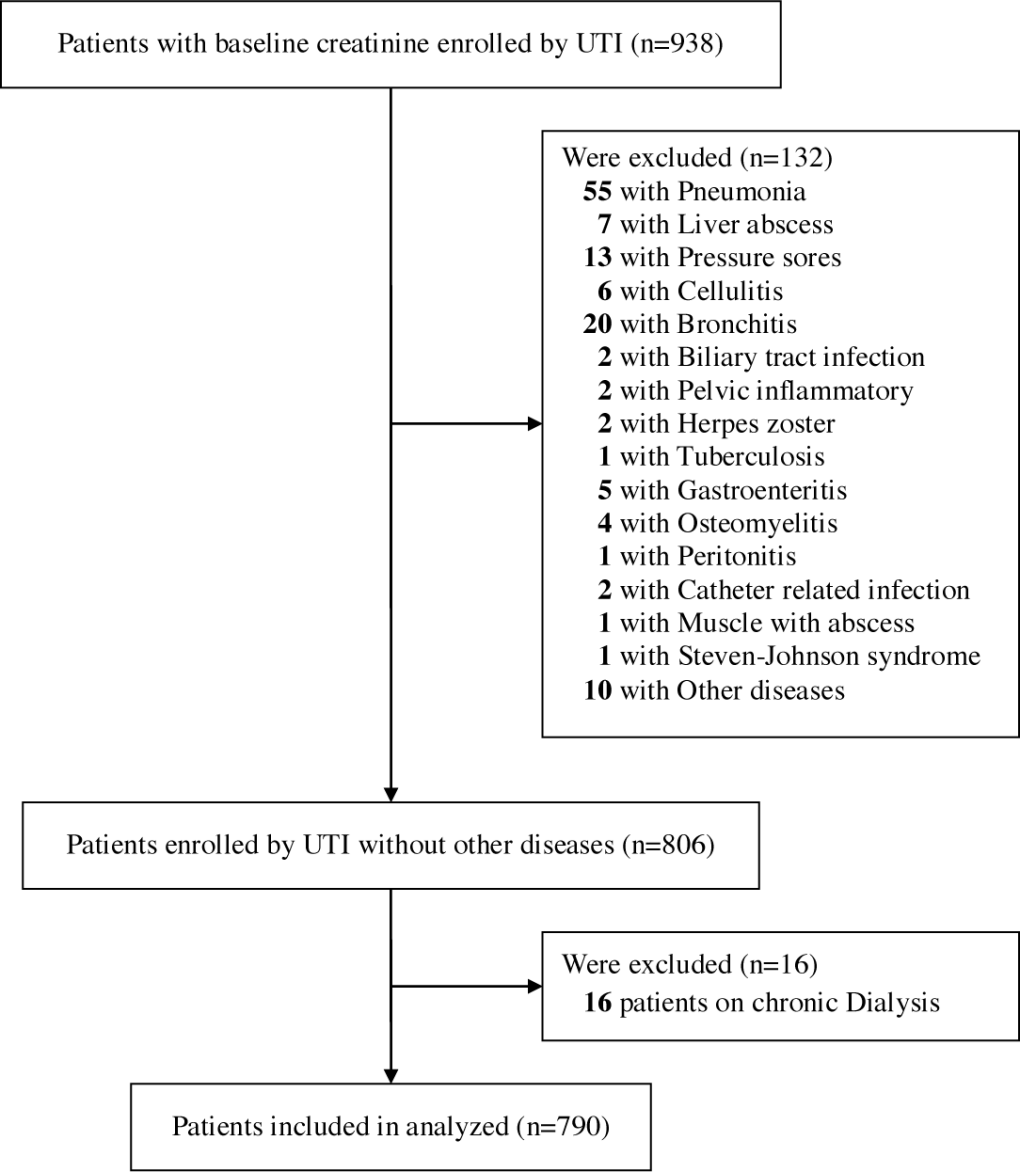
Rectangle below: patients included for analyses.

From January 2006 to January 2013, clinical data of 938 consecutive hospitalized patients with baseline creatinine values diagnosed with UTI in the Chia-Yi Christian Hospital were enrolled. The criteria for the diagnosis of UTI were symptomatic, including pain on urination, lumbago or fever with bacterial isolation of more than 10^4^ colony forming units (CFU)/mL. Patients with concurrent infections other than UTI or receiving chronic dialysis therapy (i.e., regular dialysis therapy more than 3 months) before UTI episode were excluded from this study (Fig 1). Finally a total of 790 patients with UTI were included for analyses.

### Hospital Course

Inpatients were assessed by standard laboratory and diagnostic procedures. Clinical data including age, sex, diabetes mellitus (DM), hypertension, coronary artery disease (CAD), congestive heart failure (CHF), cerebrovascular disease, malignancy, and medications such as antihypertensive drugs or nephrotoxic agents (i.e., aminoglycosides, nonsteroidal anti-inflammatory drugs (NSIAD), contrast media, and trimethoprim/sulfamethoxazole) were recorded. Patients admitted were treated with antibiotics based on the standard protocol. The initial regimens of empiric antibiotic therapy were parenteral first generation cephalosporin plus aminoglycoside (if no impaired renal function), parenteral second generation cephalosporin or parenteral fluoroquinolones to treat the common UTI pathogens for patients with stable hemodynamic condition. Parenteral empiric antibiotic therapy according to previous culture results and antimicrobial susceptibility was prescribed for patients with recurrent UTI. Specific antibiotic therapy was administered according to the culture results and antimicrobial susceptibility during hospitalization. The four main vital signs including pulse rate, respiration rate, blood pressure, and temperature were routinely monitored.

#### Major Outcomes and Definitions

AKI was diagnosed by a decrease in glomerular filtration rate (GFR) more than 50% or doubling of serum creatinine compared to that at baseline according to The RIFLE GFR criteria [14]. Estimated GFR (eGFR) was determined according to the Chronic Kidney Disease Epidemiology Collaboration (CKD-EPI) creatinine equation [15]. The baseline level of serum creatinine was obtained at dates 3 months before admission. The diagnosis and classification of chronic kidney disease (CKD) were established according to the criteria of the National Kidney Foundation K/DOQI Clinical Practice Guidelines for Chronic Kidney Disease [16]. Diagnosis of DM was based on the American Diabetes Association and the World Health Organization criteria. Upper UTI was an infection of the kidney or ureter; lower UTI included cystitis, urethritis, and prostatitis. Bacteremia was an invasion of the bloodstream by bacteria and confirmed by blood culture. Fever was defined as a temperature above 38.3°C (101°F) [17, 18]. Afebrile was defined as UTI patients who did not have a temperature above 38.3°C (101°F). Septic shock was defined as sepsis with hypotension (systolic blood pressure less than 90 mmHg or a fall in systolic blood pressure > 40 mmHg) lasting for at least 1 hour despite adequate fluid resuscitation [19].

### Study Design

Patients were divided into two groups based on the presence (group 1) or absence (group 2) of AKI during the hospital stay. Demographic data of gender, age, co-morbidities (diabetes mellitus, hypertension, CHF, CAD, stroke, and malignancy), upper or lower UTI, baseline renal function, indwelling urinary catheter, vital signs at admission, and presence of bacteremia or septic shock during hospitalization were reviewed from the medical charts and data were collected for further analyzed.

### Statistical analysis

Data are expressed as means and standard deviations for continuous variables and as frequency and proportions for categorical variables. Continuous data were analyzed using a Mann-Whitney U-test. Categorical data were analyzed by Fisher’s exact test or chi-square test. We performed a conditional logistic regression analysis with AKI as the outcome variable and baseline demography as well as clinical relevant data as the main exposure of interest. A P value under 0.05 was considered significant. Statistical analyses were performed by the software SPSS 17.0 (International Business Machines Corp., Armonk, NY, USA).

## Results

There were 790 UTI patients enrolled for final analysis, their demographic and clinical characteristics of UTI patients are shown in Table 1. The mean age was 65 ± 18 years, 543 (68.7%) were female. There were 335 hypertensive patients, among which 180 were treated with angiotensin-converting enzyme inhibitors (ACE inhibitors) or angiotensin receptor blockers (ARBs). A total of 47 patients were found to have urinary tract obstruction. A total of 369 patients were afebrile UTI during hospitalization, and 15 of them had hypothermia. There were 97 patients (12.3%) developing AKI after admission with 4 patients (0.5%) necessitating dialysis therapy. The overall mortality rate was 0.38% (3/790). Patients who developed AKI had older age (72 ± 13 versus 64 ± 19 years, *P* <0.001) and higher serum creatinine at admission (3.37 ±1.96 versus 1.32 ± 0.94 mg/dL, *P* <0.001), higher prevalence of DM (56.7% versus 35.6%, *P* <0.001), hypertension (54.6% versus 40.7%, *P* = 0.009), upper UTI (46.4% versus 35.5%, *P* = 0.037), afebrile (59.8% versus 44.9%, *P* = 0.006), septic shock (22.7% versus 9.8%, *P* <0.001) and bacteremia (39.2% versus 28.6%, *P* = 0.033), lower values of systolic and diastolic blood pressure (127 ± 33 versus 137 ± 30 mmHg, and 70 ± 17 versus 77 ± 15 mmHg, *P* = 0.006 and *P* <0.001, respectively) and baseline eGFR (53 ± 23 versus 72 ± 27 mL/min/1.73m^2^, *P* <0.001), and less exposure to nephrotoxic agents (47.4% versus 69.3%, *P* <0.001) compared with those without AKI.

**Table 1:** Characteristics of the 790 patients with urinary tract infection.

Characteristic	All (n = 790)	Acute kidney injury		*P*-Value
		Yes (n = 97)	No (n = 693)	
Age (year)	65 ± 18	72 ± 13	64 ± 19	<0.001
Gender (female)	543 (68.7)	72 (74.2)	471 (68.0)	0.213
Systolic blood pressure (mmHg)	135 ± 30	127 ± 33	137 ± 30	0.006
Diastolic blood pressure (mmHg)	75.82 ± 15.8	70 ± 17	77 ± 15	<0.001
Diabetes mellitus	302 (38.2)	55 (56.7)	247 (35.6)	<0.001
Hypertension	335 (42.4)	53 (54.6)	282 (40.7)	0.009
Congestive heart failure	29 (3.7)	5 (5.2)	24 (3.5)	0.387
Coronary artery disease	55 (7.0)	10 (10.3)	45 (6.5)	0.167
Stroke	178 (22.5)	21 (21.6)	157 (22.7)	0.824
Malignancy	92 (11.6)	16 (16.5)	76 (11.0)	0.112
Indwelling foley catheter	54 (6.8)	8 (8.2)	46 (6.6)	0.556
Afebrile	369 (46.7)	58 (59.8)	311 (44.9)	0.006
Upper urinary tract infection	291 (36.8)	45 (46.4)	246 (35.5)	0.037
Bacteremia	236 (29.9)	38 (39.2)	198 (28.6)	0.033
Septic shock	90 (11.4)	22 (22.7)	68 (9.8)	<0.001
Hospitalized serum creatinine (mg/dL)	1.57 ± 1.30	3.37 ± 1.96	1.32 ± 0.94	<0.001
Baseline eGFR (mL/min/1.73 m^2^)	70 ± 28	53 ± 23	72 ± 27	<0.001
Baseline renal function (eGFR)				<0.001
≥ 60 mL/min/1.73m^2^	497 (62.9)	32 (33.0)	465 (67.1)	
45–59 mL/min/1.73m^2^	132 (16.7)	23 (23.7)	109 (15.7)	
30–44 mL/min/1.73m^2^	103 (13.0)	27 (27.8)	76 (11.0)	
< 30 mL/min/1.73m^2^	58 (7.3)	15 (15.5)	43 (6.2)	
Nephrotoxic agents	526 (66.6)	46 (47.4)	480 (69.3)	<0.001
Aminoglycosides	290 (36.8)	16 (16.3)	274 (39.7)	<0.001
Nonsteroidal anti-inflammatory drugs	392 (49.6)	36 (36.7)	356 (51.4)	0.006
Contrast media	99 (12.5)	16 (16.3)	83 (12.0)	0.225
Trimethoprim/sulfamethoxazole	21 (2.7)	4 (4.1)	17 (2.5)	0.317
Antihypertensive agents	429 (54.3)	66 (68.0)	363 (52.4)	0.004

Data are expressed as mean ± SD or number (percentage)

**Fig 2:**
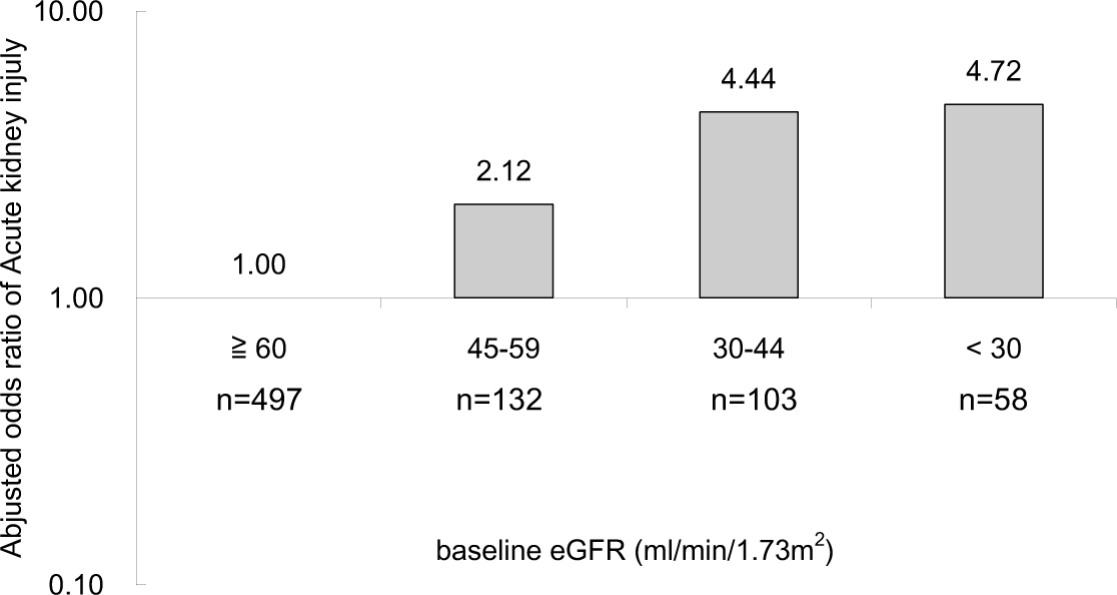
Multivariate analysis of associations between eGFR and acute kidney injury. ^a^N = 790. ^b^Multivariate model adjusted for gender, diabetes mellitus, hypertension, congestive heart failure, coronary artery disease, stroke, malignancy, indwelling foley catheter, afebrile, upper UTI, septic shock, baseline eGFR group.

In multivariate logistic regression analysis, older age (OR 1.02, 95% CI 1.00–1.04, *P* = 0.04), DM (OR 2.23, 95% CI 1.35–3.68, *P* = 0002), upper UTI (OR 2.63, 95% CI 1.53–4.56, *P* = 0001), afebrile during hospitalization (OR 1.71, 95% CI 1.04–2.83, *P* = 0036) and lower baseline eGFR [baseline eGFR 45–59 mL/min/1.73 m^2^ (OR 2.12, 95% CI 1.12–4.04, *P* = 0.022), baseline eGFR 30–44 mL/min/1.73 m^2^ (OR 4.44, 95% CI 2.30–8.60 *P* < 0.001) baseline eGFR < 30 mL/min/1.73 m^2^ (OR 4.72, 95% CI 2.13–10.45, *P* <0.001), respectively] were independently associated with increased risk for development of AKI in patients admitted with UTI (Table 2) (Fig 2).

**Table 2:** Multivairate logistic regression model for factors related to acute kidney injury.

Covariate	*β*	OR (95% CI)	*P*-Value
Age (year)	0021	1.02 (1.00–1.04)	0040
Gender (female)	0280	1.32 (0.77–2.27)	0307
Systolic blood pressure (mmHg)	-0010	0.99 (0.98–1.00)	0079
Diastolic blood pressure (mmHg)	-0015	0.99 (0.96–1.01)	0165
Diabetes mellitus	0801	2.23 (1.35–3.68)	0002
Hypertension	0172	1.19 (0.71–1.99)	0515
Congestive heart failure	-0397	0.67 (0.20–2.21)	0513
Coronary artery disease	-0033	0.97 (0.42–2.21)	0937
Stroke	-0216	0.81 (0.44–1.49)	0490
Malignancy	0325	1.38 (0.71–2.68)	0337
Indwelling foley catheter	0294	1.34 (0.56–3.23)	0513
Afebrile	0538	1.71 (1.04–2.83)	0036
Upper urinary tract infection	0970	2.63 (1.53–4.56)	0001
Septic shock	0552	1.74 (0.89–3.38)	0104
Baseline eGFR group			
45–59 versus 60 ml/min/1.73 m^2^	0.753	2.12 (1.12–4.04)	0.022
30–44 versus 60 ml/min/1.73 m^2^	1.491	4.44 (2.30–8.60)	<0.001
< 30 versus 60 ml/min/1.73 m^2^	1.552	4.72 (2.13–10.45)	<0.001

## Discussion

AKI is a common complication of sepsis and septic shock. UTI is one of the common causes of sepsis and may cause sudden deterioration in renal function. Several studies suggested that AKI was not a common complication among patients with acute pyelonephritis [20–22]. In this study, the incidence of AKI in UTI patient necessitating admission was 12.3%, with a bacteremia rate of 29.9%, septic shock 11.4%, and mortality 0.38%. Only a few studies investigated the risk factors for AKI in UTI patients. Previous studies indicated that hypovolemia, hypotension, sepsis, the use of nephrotoxic drugs, contrast media and urinary obstruction were AKI risk factors in UTI patients [23, 24]. Our study showed that UTI patients with DM, upper UTI, afebrile or septic shock during hospitalization and impaired baseline renal function were at higher risk for development of AKI.

The risk of UTI in CKD patients might be increased by disease and host factors (e.g., papillary necrosis, nephrolithiasis, neurogenic bladder, immunodeficiency, malnutrition, low urinary flow rate or urinary concentration defect) and management of comorbidity (foley catheters and intravenous lines) [25]. In addition, CKD has been recognized as a risk factor for development of AKI. A lot of comorbidities are associated with CKD, including high incidence of cardiovascular disease in CKD with increased exposure to contrast agents, use of ACE inhibitors or ARBs in the existence of undiagnosed renal artery stenosis, and impaired autoregulation of renal blood flow in diabetic patients permitting low renal perfusion during systemic hypotension. These comorbidities *per se* may lead to more frequent exposure to nephrotoxic agents and/or alter the response to an acute insult, which cause increased susceptibility to AKI [26]. Previous studies reported that CKD was a potent predictor of acute decline in kidney function following exposure to radio-contrast and major surgery [27, 28]. Hsu *et al*. compared CKD patients with hospital-acquired AKI treated with and without dialysis, and identified a significantly increased AKI risk if eGFR <60 mL/min/1.73 m^2^ [29]. Moreover, subjects with eGFR of 45–59 mL/min/1.73 m^2^ had on average a twofold increase in adjusted odds ratio of AKI compared with subjects with eGFR of 60 mL/min/1.73 m^2^ or above. In this study, there was a higher incidence of eGFR of <60 mL/min/1.73 m^2^ in the AKI group compared with that in the non-AKI group (67.0% versus 32.9%, respectively). In addition, compared with subjects with baseline eGFR of 60 mL/min/1.73 m^2^ or above, the odds ratio for AKI in subjects with baseline eGFR 45–59 mL/min/1.73 m^2^, 30–44 mL/min/1.73 m^2^, and < 30 mL/min/1.73 m^2^ were 2.12, 4.44, and 4.72, respectively. Our results suggested that impaired baseline renal function played a predictive role for development of AKI in UTI patient.

Our study showed that the UTI patients with AKI were older than those without (72 ± 13 versus 64 ± 19 years, P < 0.001), with an odds ratio of 1.02 increment each year. UTI is a type of infection which is common among older people. The prevalence of UTI in the elderly is much higher than younger individuals. At least 20% women and 10% of men aged 65 years or older have bacteriuria [30]. In our study, 59.2% (468/790) of the cases are over 65 years old. Multiple age-related changes including cell-mediated immunity recession, bladder defenses alteration due to obstructive uropathy, neurogenic dysfunction, bacterial receptivity intensification of uroepithelial cells [31], contamination due to fecal and urinary incontinence, uretheral instrumentation and catheterization, and antibacterial factors reduction in prostate and vagina associated with changes in zinc levels, PH and hormones [32] contribute to the risk associated with UTI in elderly. Additionally, age-related decline of GFR may cause older patients at risk for acute kidney injury. [33] In our study, patients who were over 65 years old had higher prevalence of eGFR <60 compared with those under 65 years old (53.2% versus 13.7%, respectively). Since age and impaired baseline renal function are risks for development of AKI in UTI patients, physicians should pay attention to elderly UTI patients with CKD.

DM has been reported as a risk factor for the development of both upper and lower UTIs [34–36]. In addition, DM is a potent predictor of AKI following exposure to contrast media [27], cardiac surgery [37, 38], oral sodium phosphate bowel preparation [39], and sepsis [40]. In our study, UTI patients, compared with the general population, had a higher incidence of DM (38.2%). In the AKI group, 56.7% had DM. Patients with diabetes had a higher risk of AKI (OR 2.23, 95% CI 1.35–3.68, *P* = 0.002). UTI is one of the most common sources of bacteremia in diabetic patients [41], and patients with DM are at a greater risk of developing various complications of UTI including sepsis [42]. Severe sepsis may induce vital organ dysfunction, including AKI [43]. Robbins *et al*. showed that UTI induced acute kidney injury in approximately 40% of diabetic patients with bacteremia [44]. Our results also suggested that DM was an independent risk factor for AKI in UTI patients.

Fever is known as an important feature of sepsis, and is considered to be an adaptive response to strengthen the immune system in order fighting against the invading organisms [45]. Lack of fever may contribute to lower resistance to infection, delayed recovery [17], higher mortality rate, and poor prognosis [46]. A number of factors, including acute alcoholism, hypothyroidism and elderliness have been identified in patients who had experienced afebrile bacteremia [47–49]. In this study, there was a higher incidence of being afebrile in patients with AKI (59.8%) than those without AKI (44.9%). Being afebrile played a predictive role for AKI in UTI patient (OR 1.71, 95% CI 1.04–2.83, *P* = 0.036). Wolk PJ *et al*. found that patients with renal impairment exhibited a lower febrile response to bacteremia [50]. Our study showed that 50.4% (137/272) of patients with eGFR <60 mL/min/1.73m^2^ experienced afebrile UTI. Among the UTI patients with eGFR <60 mL/min/1.73m^2^ developing AKI, 64.5% (40/62) presented with being afebrile. Several reasons might account for afebrile UTI patients being prone to developing AKI. First, patients with afebrile UTI have higher chance to have CKD, whereas CKD *per se* is a risk of AKI in UTI patients. Second, afebrile UTI with sepsis is more likely to be unrecognized, and more serious complications including AKI may result from the delayed treatment.

Upper UTI is not a well-recognized cause of AKI. Acute pyelonephritis can involve entire lobules of the medulla and cortex [51]. Interstitial infiltration of neutrophils and phagocytes and extensive destruction of the parenchymal by the acute inflammatory process were found in AKI patients with acute pyelonephritis [52. 53]. These reports demonstrated that severe upper UTI might cause serious damage to the kidney and resulted in AKI. Our study found that patients with upper UTI had higher risk of AKI than those with lower UTI (OR 2.63, 95% CI 1.53–4.56, *P* = 0.001). Early institution of appropriate antibiotic therapy for patients with upper UTI, especially those with septic shock, is important to prevent the development of AKI.

It is well known that nephrotoxic medications contribute to the development of AKI [54, 55]. In this study, there was a lower incidence of AKI in patients treated with nephrotoxic agents compared with those not treated with nephrotoxic agents (8.7% versus 19.7%, respectively). Nephrotoxic agent was usually avoided in patients with impaired baseline renal function, unstable hemodynamics, or at risk of AKI. This would result in a lower incidence of use of nephrotoxic agents in patients developing AKI in this study.

There are several limitations in this study. First, the retrospective data collection may lead to missing data and bias. However, we comprehensively collected the data using a standard form to reduce bias. Second, although the treatments were prescribed by qualified attending physicians according to the standard treatment for UTI, medications potentially contribute to AKI (e.g., aminoglycosides, nonsteroid anti-inflammatory drugs, contrast media), which may result in a potential bias. Nephrotoxic agents were usually avoided in patients with impaired baseline renal function or at risk of AKI. Contrast media may be required for emergency imaging survey of complicated UTI, urosepsis, or septic shock. Because of the selection bias, putting the use of nephrotoxic agent into the multivariate analysis will yield inaccurate results or conclusions. Further prospective studies will be needed to confirm the relationship between nephrotoxic agents and AKI in UTI patients. Third, this is a single center study, and the results may not be generalized to the entire community. A prospective, randomized study with larger sample size will be needed to confirm our results. Fourth, UTI patients’ urine output was not routinely recorded in this retrospective study. Therefore, we could not use the definition of AKI based on the urine output of Kidney Disease: Improving Global Outcomes (KDIGO) criteria to define the development of AKI. Of course, this underestimated the true incidence of AKI in our UTI patients. A prospective study designed to record the serial serum creatinine and urine output after admission will be needed to provide the true incidence of AKI after UTI using the KDIGO criteria based on both serum creatinine and urine output criteria. In conclusion, we found that impaired baseline renal function, advancing age, DM, upper UTI, and afebrile during hospitalization are all important and independent risk factors for developing AKI in UTI patients. Physicians should pay attention to UTI patients at risk of AKI.

## Supporting Information

S1 Dataset Data of patient with urinary tract infection. (XLS)
